# The Teaching of Homœopathy in the Colleges

**Published:** 1891-01

**Authors:** Edward F. Brady

**Affiliations:** Professor Materia Medica Kansas City Homœopathic Medical College; Kansas City, Mo.


### THE TEACHING OF HOMŒOPATHY IN THE COLLEGES.

KANSAS CITY, MO., December 10th, 1890.

EDITORS HOMŒOPATHIC PHYSICIAN :

The returns are not all in yet on that very important subject, the teaching of pure Homœopathy in our colleges. You will have to still further qualify your statements made in the October number of the journal. As the Professor of Materia Medica in the Kansas City Homœopathic College, I most emphatically protest against your statement. I, myself, am a thoroughgoing Hahnemannian homœopath; the *Organon* is my medical Bible, and in so far as I am able I inculcate the mighty truths taught in its pages. Class-reading of the *Organon* is a part of the lecture course. Students for graduation have been notified that examination questions from this chair in part will be asked from and bearing directly on principles laid down in that book.

Requesting as wide a circulation for the foregoing as was extended your editorial comment,

I am, fraternally yours,

EDWARD F. BRADY,

Professor Materia Medica Kansas City Homœopathic Medical College.

